# Loss of Imprinting and Allelic Switching at the *DLK1-MEG3* Locus in Human Hepatocellular Carcinoma

**DOI:** 10.1371/journal.pone.0049462

**Published:** 2012-11-08

**Authors:** Sumadi Lukman Anwar, Till Krech, Britta Hasemeier, Elisa Schipper, Nora Schweitzer, Arndt Vogel, Hans Kreipe, Ulrich Lehmann

**Affiliations:** 1 Institute of Pathology, Medizinische Hochschule Hannover, Hannover, Germany; 2 Department of Gastroenterology, Hepatology and Endocrinology, Medizinische Hochschule Hannover, Hannover, Germany; University of Hong Kong, Hong Kong

## Abstract

Deregulation of imprinted genes is an important molecular mechanism contributing to the development of cancer in humans. However, knowledge about imprinting defects in human hepatocellular carcinoma (HCC), the third leading cause of cancer mortality worldwide, is still limited. Therefore, a systematic meta-analysis of the expression of 223 imprinted loci in human HCC was initiated. This screen revealed that the *DLK1-MEG3* locus is frequently deregulated in HCC. Deregulation of *DLK1* and *MEG3* expression accompanied by extensive aberrations in DNA methylation could be confirmed experimentally in an independent series of human HCC (n = 40) in more than 80% of cases. Loss of methylation at the *DLK1-MEG3* locus correlates linearly with global loss of DNA methylation in HCC (r^2^ = 0.63, p<0.0001). Inhibition of DNMT1 in HCC cells using siRNA led to a reduction in *MEG3-DMR* methylation and concomitant increase in *MEG3* RNA expression. Allele-specific expression analysis identified loss of imprinting in 10 out of 31 informative samples (32%), rendering it one of the most frequent molecular defects in human HCC. In 2 cases unequivocal gain of bi-allelic expression accompanied by substantial loss of methylation at the *IG-DMR* could be demonstrated. In 8 cases the tumour cells displayed allelic switching by mono-allelic expression of the normally imprinted allele. Allelic switching was accompanied by gains or losses of DNA methylation primarily at *IG-DMR1*. Analysis of 10 hepatocellular adenomas (HCA) and 5 cases of focal nodular hyperplasia (FNH) confirmed that this epigenetic instability is specifically associated with the process of malignant transformation and not linked to increased proliferation *per se*. This widespread imprint instability in human HCC has to be considered in order to minimize unwanted side-effects of therapeutic approaches targeting the DNA methylation machinery. It might also serve in the future as predictive biomarker and for monitoring response to epigenetic therapy.

## Introduction

The development of cancer in humans is not only caused by genetic lesions (mutations, deletions, translocations etc.) [1], but also by epigenetic aberrations [2]. One epigenetic phenomenon whose deregulation contributes to the development and progression of cancer in humans is imprinting, the parent-of-origin specific expression of genes. In the human genome about 200 genes are imprinted [3], displaying preferential expression of one allele or even strict mono-allelic expression.

A causal role of imprinting aberrations in human carcinogenesis is suggested by several human disorders, e.g., complete parthenogenesis in ovarian teratomas [4] and androgenic conception in hydatidiform moles [5]. Contribution of imprinting defects in cancer is best exemplified in patients with Beckwith-Wiedemann syndrome (BWS) [6], [7]. Deregulation of imprinted genes in the 11p15.5 imprinting locus caused by mutations, epimutations, or uniparental inheritance affects proliferation control in BWS patients predisposing them with a 600 fold increase in cancer risk, especially for embryonic tumours such as Wilms' tumour or hepatoblastoma [8].

Since some genes demonstrate developmental stage-specific or tissue specific imprinting [9], the study of imprinting can be complicated and the comparison of results from different studies might be misleading. The use of proper controls for the identification of imprint alterations is of uppermost importance. These experimental challenges (see also [10]) might be the reason why much less is known about imprinting defects in human cancer compared to aberrant promoter hypermethylation of tumour suppressor genes, despite the fact the imprinting defects in tumour cells have actually been described several years before the identification of tumour suppressor gene hypermethylation [11], [12].

This holds true especially for human hepatocellular carcinoma (HCC), the third leading cause of cancer mortality worldwide [13]. Only 11 publications could be identified in PubMed (from 1991 to June 2012) addressing specifically the allele-specific DNA methylation and/or expression of imprinted loci in primary human HCC specimens (excluding all animal and cell line studies) [14], [15], [16], [17], [18], [19], [20], [21], [22], [23], [24]. Since the majority of these studies (7 of 11) concentrate on a single locus, i.e., *IGF2/H19*, we started a systematic *in silico* analysis of the expression of all known imprinted loci in human HCC in order to identify imprinted loci deregulated in human HCC. After validation of candidates in our own cohort, we concentrated on the analysis of the *DLK1-MEG3* imprinting locus on chromosome 14q32 which is frequently deregulated in several paediatric tumours [25] and reported to have tumour suppressor activities [26], [27].

At the start of the project (end of 2010) only a single publication about *MEG3* expression in human HCC could be identified (reporting no alteration in *MEG3* expression in 10 HCC samples, [15]). Recently, another study analysing MEG3 expression in a small series of human HCC was published [14] which is analysed in detail in the “Discussion” section.

After screening published expression data sets for deregulated imprinted loci in human HCC we could show that the expression of the *DLK1/MEG3* locus is deregulated in more than 80% of human HCC accompanied by extensive aberrations in DNA methylation.

## Results

### Identification of imprinted loci deregulated in human HCC

**Figure 1:**
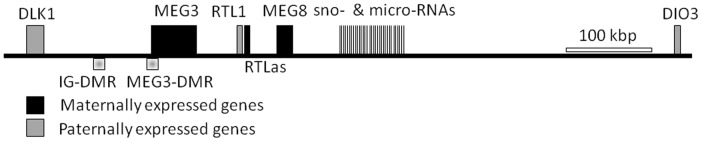
Schematic representation of the *DLK1-MEG3* imprinted locus on chromosome 14q32. Maternally expressed genes are depicted in black and paternally expressed genes in grey. The intergenic differentially methylated region (*IG-DMR*) is located approximately 14 kb upstream to the first exon of *MEG3*. Studies in patients with UPD chromosome 14 revealed two regions with differential methylation at IG-DMR [30]. Therefore two pyrosequencing assays were developed at this region (*IG-DMR*1 and 2). In addition, three pyrosequencing assays were designed for one CpG island and two CTCF binding sites within the *MEG3*-DMR which was found to display parent-of-origin specific methylation (*MEG3*-DMR1, 2, and 3). Horizontal bar represents scale of 100 kbp.

Using expression profiles deposited in the database Oncomine [28] 223 imprinted loci of the human genome were screened for deregulated expression in human HCC. The comprehensive list of imprinted loci was retrieved from the databases “Geneimprint” (http://www.geneimprint.org/) and “A Catalogue of Parent-of-Origin Effects” (www.otago.ac.nz/IGC). Within Oncomine a set of 16 expression profiles comprising altogether 953 primary human HCC specimens were identified and evaluated (Table S1). From these datasets, we identified 26 imprinted genes as down-regulated and 12 genes as up-regulated in primary human liver tumour samples and/or HCC cell lines (see Table S2). Subsequent analyses focussed on the non-coding RNA *MEG3*, because it showed the most frequent deregulation of expression in primary HCC according to this meta-analysis and also in an initial screen performed by ourselves (see below). Since *MEG3* is part of the *DLK1-MEG3* imprinting locus (see Figure 1), the expression and regulation of *DLK1* was also analysed in this study.

### Deregulation of *DLK1* and *MEG3* expression in human HCC

**Figure 2:**
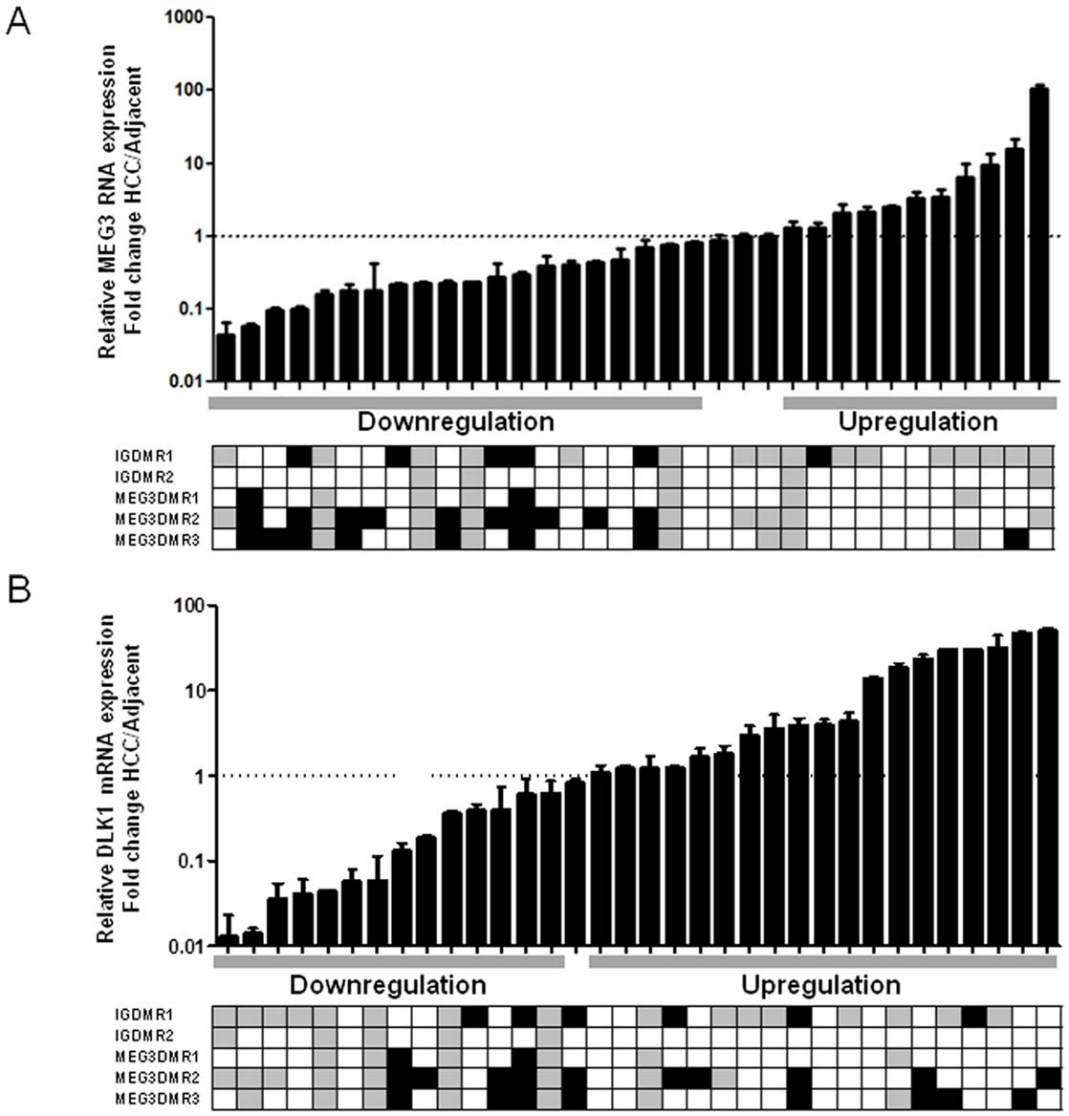
Expression and DNA methylation of *MEG3* RNA and *DLK1* mRNA in primary human HCC. *MEG3* RNA (A) and *DLK*1 mRNA (B) expression was measured in a series of 34 paired HCC using quantitative RT-PCR. For every sample, the RNA and mRNA levels were normalized to the mean expression of *GUSB* and *GAPDH*. Expression in primary HCC samples was then normalized to the corresponding adjacent non-cancerous liver tissues. Relative expression is presented as fold change in a logarithmic scale. Samples were classified as “up-regulated” if the lower limit of the 98% confidence interval was larger than 1 and “down-regulated” if the upper limit was smaller than 1. DNA methylation levels were measured using high-resolution quantitative pyrosequencing and are displayed below the expression data as hypermethylated (black box), hypomethylated (light grey) or normomethylated (white box). For definition of thresholds see Materials and Methods. The computed methylation levels are the average of two independent pyrosequencing runs. The pyrosequencing assays for *IG-DMR*1, *IG-DMR*2, and *MEG3-DMR*1 contain 5 CG sites, for *MEG3-DMR*2 9 CG sites, for *MEG3-DMR3* 6 CG sites. A positive association of high methylation with reduced *MEG3* expression (panel A), left part) and reduced methylation and low *DLK1* expression (panel B), left part) is clearly visible.

The expression of *MEG3* and *DLK1* was analysed in a series of 34 primary human HCC specimens and the corresponding adjacent liver tissue samples using quantitative real-time PCR. This revealed frequent and extensive deregulation in *MEG3* RNA and *DLK1* mRNA expression (Figure 2): 20 HCC samples display a *MEG3* down-regulation (59%), whereas 11 samples show an increase in expression (32%). *DLK1* mRNA is increased in 18 (53%) and reduced in 15 cases (44%).

### DNA methylation patterns at the *DLK1-MEG3* imprinting locus in human HCC

Since the *DLK1-MEG3* locus displays imprinting and mono-allelic expression [29], [30], [31], the loss or gain of DNA methylation as a cause of deregulated expression was studied. In a panel of established HCC cell lines frequent and extensive gain or loss of DNA methylation at this locus could be demonstrated using newly established pyrosequencing assays (see Figure S1). In line with these finding also primary HCC specimens display frequent and extensive alterations in DNA methylation patterns (Fig. 2 and Figure S2). If all differentially methylated regions (DMRs) under study are considered together, 33 out of 40 samples display aberrations in DNA methylation (82.5%, Fig. 2)

**Figure 3:**
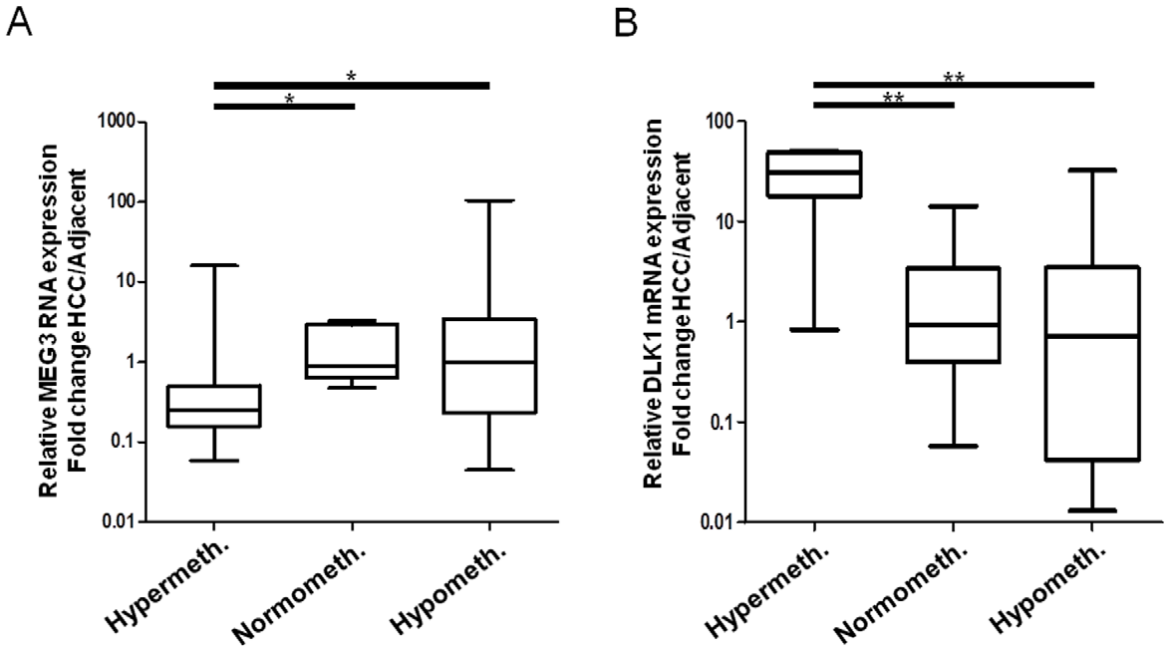
Relationship between DNA methylation and gene expression at the *DLK1-MEG3* locus. Primary HCC samples are defined as “hypermethylated” and “hypomethylated” using mean ±2×SD of the methylation levels of the adjacent non-cancerous liver tissues. Relative *MEG3* and *DLK1* expression is then compared among hyper-, normo-, and hypo-methylated subgroups. The horizontal lines inside the box represents the median of relative *MEG3* expression and the upper and lower edges of each box represent the 75th and 25th percentile respectively, while the bars denote the highest and lowest relative expression measured. * = 0.001<p<0.05, ** = 0.0001<p<0.001.

If the HCC samples are sorted according to their methylation status (i.e., hypomethylated, hypermethylated, and normomethylated, for threshold definition see “Materials and Methods”) the negative correlation between the methylation at the *DLK1/MEG3-DMR*s and the expression of the *MEG3* RNA becomes obvious (Figure 3 A), Fisher's exact test: p = 0.018). By contrast methylation at the *DLK1/MEG3-DMR*s is positively correlated with *DLK1* mRNA expression level (Figure 3 B), Fisher's exact test: p = 0.006). This relation between an increase in DNA methylation and reduction in *MEG3* expression as well as a decrease in DNA methylation and a decrease in *DLK1* expression is also obvious from Figure 2.

### Inhibition of DNA methylation restores *MEG3* expression in HCC cells

**Figure 4:**
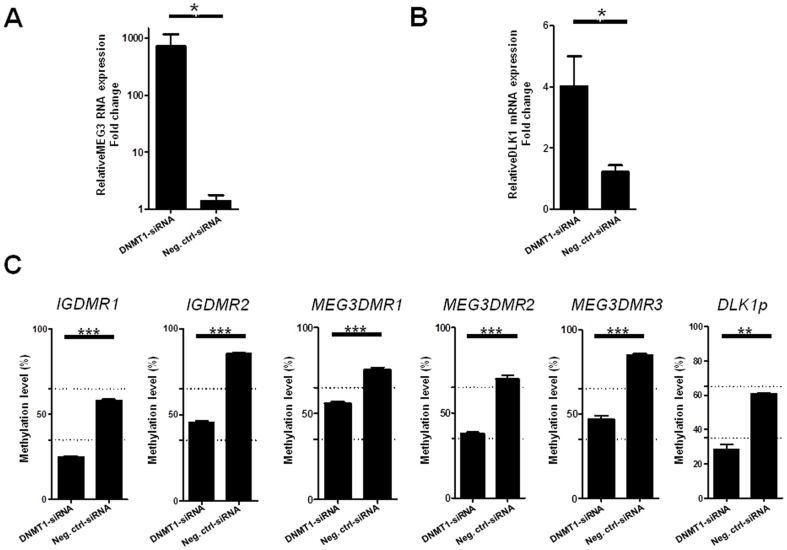
Restoration of *MEG*3-RNA and *DLK*1 mRNA expression after *DNMT*1 knock down and reduction of methylation at *MEG3* imprinting loci. (A) Quantitative *MEG3* RNA and (B) *DLK*1 mRNA expression levels in HLE cells after *DNMT*1 knockdown after normalization to *GUSB* and *GAPDH* levels (p = 0.029). (C) Reduction of DNA methylation at *DLK1/MEG3* imprinting locus after *DNMT*1 knock down (*DLK1p*: *DLK1* promoter). Methylation analysis was performed using quantitative pyrosequencing. Paired t-test showed significant decrease of methylation after *DNMT*1 knockdown for all six regions. Values shown for qRT-PCR and DNA methylation analysis are means from two independent DNMT1-knockdown experiments (50 nM and 100 nM) and two different negative control siRNAs. * = 0.001<p<0.05, *** = p<0.0001.

To elucidate the effect of DNA methylation on the expression of *MEG3*-RNA in more detail we employed inhibition of DNA methyltransferase 1 (*DNMT1*) and subsequent quantification of *MEG3*-RNA expression and methylation of the *DLK1-MEG3* locus. Figure 4 A) demonstrate that the *DNMT1* knockdown in HLE cells is able to induce re-expression of *MEG3* accompanied by significant loss of DNA methylation at *IG*- and *MEG3-DMR* (Fig. 4 C), p<0.0001). The effectiveness of the siRNA-mediated knock-down of *DNMT1* mRNA and protein in the HCC cell line HLE is shown in Figure S3.

### Deregulated *DLK1-MEG3* methylation and global methylation level in human HCC

In order to study the relationship between the methylation at the *DLK1-MEG3* locus and the global DNA methylation level in these tumour specimens, the latter was measured using the methylation level of the repetitive sequence *LINE-1* as surrogate marker for global DNA methylation [32].

**Figure 5:**
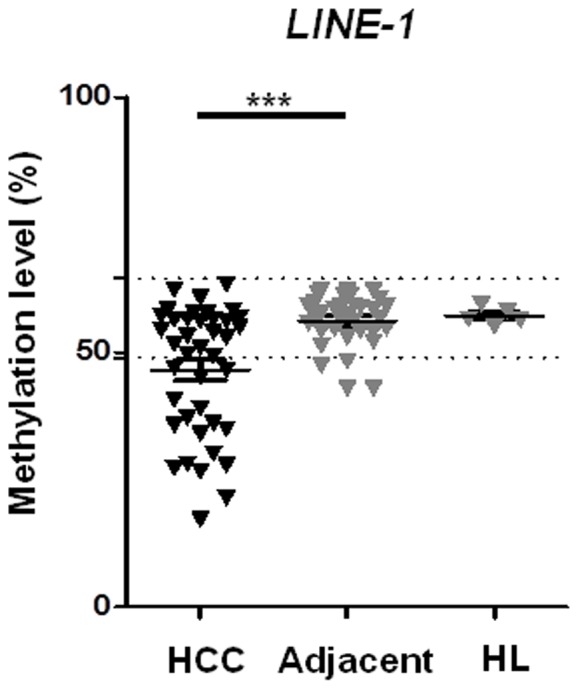
Global DNA methylation level in primary human liver specimens. The global DNA methylation level in primary HCC samples, the corresponding adjacent non-cancerous liver tissues, as well as in unrelated healthy liver tissue was determined employing quantitative pyrosequencing of *LINE1* sequences as described [40]. Methylation levels at *LINE-1* locus were significantly lower in primary HCC samples compared to the corresponding adjacent non-cancerous liver tissues. Dashed lines represent cut off values defining hyper- or hypomethylation (mean+/−2× std).

Average *LINE-1* DNA methylation was significantly lower in primary HCC samples (46%±12.6%) compared to the corresponding adjacent non-cancerous liver tissue (56.7%±8.8%), (see Figure 5), p = 0.0002). The mean global methylation level of healthy liver specimens (n = 5) was nearly identical to the mean value of the liver tissues adjacent to a tumour (57.1%).

The quantitative methylation levels at the *IG-DMR*s and the *MEG3-DMRs* in human specimens are overall linearly correlated with the global methylation level within these specimens (r^2^ = 0.24–0.63, p = 0.0012 and smaller, see figure S4). However, in several instances gain of methylation occurs in the context of global loss of methylation (e.g., #9 and #19 in Fig. 2).

### DNA methylation at the *DLK1-MEG3* imprinting locus in benign liver tumours

Addressing the question whether the above described aberrations in imprinting are specific for *bona fide* malignant proliferations and not associated with proliferation of hepatocytes *per se* DNA methylation patterns at the *DLK1-MEG3* locus were analysed in hepatocellular adenoma (HCA, n = 10) and in focal nodular hyperplasia (FNH, n = 5) using quantitative pyrosequencing. HCA show a very limited propensity for progressing to overt malignancy [33] whereas FNH represents a clearly benign liver cell proliferation [34]. Neither showed any alteration in DNA methylation at the *DLK1-MEG3* imprinting locus (figure S5). Also the global methylation level (as measured by LINE-1 methylation value) was not altered in HCA and FNH compared to adjacent liver tissue and compared to unrelated healthy liver (n = 5, figure S6).

### Deregulated *DLK1-MEG3* methylation leads to bi-allelic expression or allelic switching in human HCC

In order to analyse the impact of aberrant DNA methylation on the imprinting status of the *DLK1-MEG3* locus, allele-specific expression analyses were performed. For this purpose SNPs located within exon 3 and exon 6 of the *MEG3* gene as well as one SNP within exon 5 of the *DLK1* gene as reported by Misyoshi et al. [31] and Kagami et al. [29] were analysed for the identification of informative specimens. Sequence analysis using genomic DNA from 40 primary HCC tumours revealed 16 specimens informative for *MEG3* and 24 specimens informative for *DLK1* (in total 31 informative cases, see Table S4 for details).

**Figure 6:**
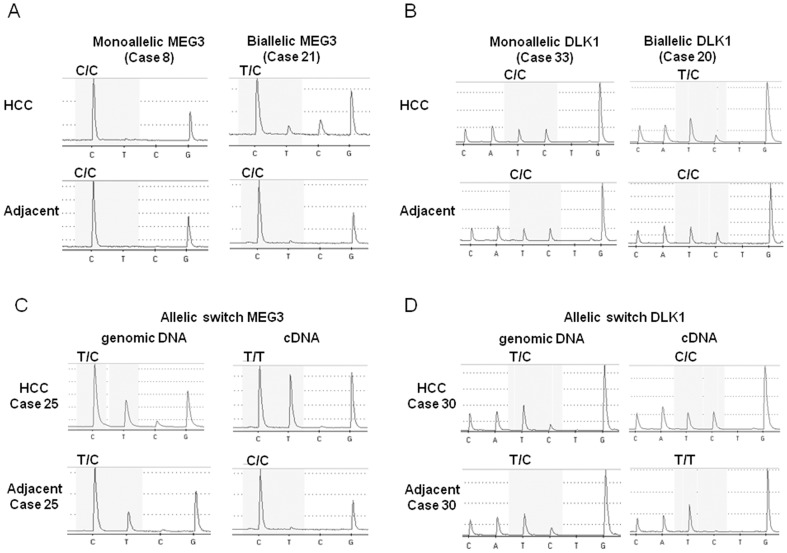
Allele-specific expression analysis of the *DLK1-MEG3* locus in human HCC. Quantitative SNP analysis was performed using pyrosequencing at a C/G polymorphism in exon 3, at SNP Rs8013873 for *MEG*3 and SNP Rs1802710 for *DLK*1. In tumour specimens showing genomic DNA polymorphisms, further SNP analysis was performed with the cDNAs from the same sample as well as with genomic and cDNA from the corresponding adjacent non-cancerous liver tissues. (A) Pyrosequencing analysis of the cDNA at SNP Rs8013873 shows mono-allelic *MEG*3 expression in HCC specimen #8 and bi-allelic expression in HCC specimen #21. (B) Pyrosequencing analysis of the cDNA at SNP Rs1802710 shows mono-allelic *DLK*1 expression in HCC specimen #33 and bi-allelic expression in HCC specimen #20. (C) *MEG*3 allelic switching is shown in case #25. Informative polymorphisms are found in the genomic DNAs of both tumour and the adjacent non-cancerous tissues and T/T expression in cDNA tumour but C/C expression in cDNA adjacent liver tissues. (D) *DLK*1 allelic switching is shown in case #30 with informative polymorphisms in the genomic DNAs of both tumour and the adjacent non-cancerous tissues and C/C expression in tumour cDNA but T/T expression in cDNA from the adjacent liver tissues. The confirmation of all genotyping results by Sanger sequencing is shown in Figure S5.

Quantitative SNP analysis of cDNA preparations from heterozygous samples employing pyrosequencing showed unequivocally bi-allelic *MEG3* expression in one case and bi-allelic *DLK1* expression in another case (Figure 6 A) and B)). These results were confirmed by Sanger sequencing (see Figure S7, panel A). The corresponding adjacent liver tissue displays mono-allelic expression in both cases. In addition to this well described loss of imprinting, 8 heterozygous samples displayed allelic switching (2 for *MEG3*, 6 for *DLK1*, see Figure 6 C) and D) as well as figure S8). Since in all 8 cases the genomic DNA from the tumour is clearly heterozygous (with the expected 50∶50 ratio, see Figure S8) this switch in the expressed allele cannot be due to relaxation of imprinting (resulting in bi-allelic expression) and subsequent loss of the originally imprinted allele but is caused by switching of the expressed allele. Therefore, altogether 32% (10 out of 31) of informative HCC specimens from our cohort display imprinting aberrations in the *DLK1-MEG3* locus.

### Changes in DNA methylation patterns underlying allelic switching at the *DLK1-MEG3* locus in human HCC

**Figure 7:**
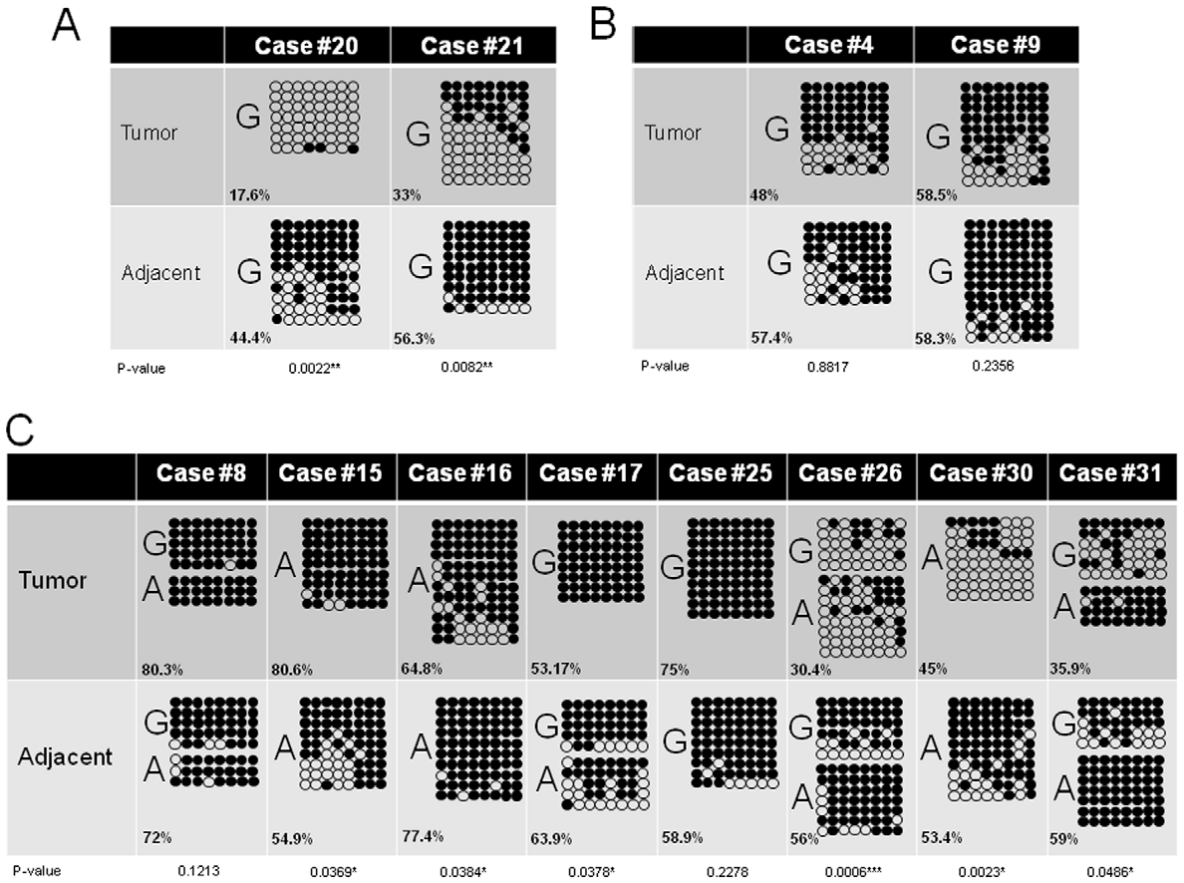
DNA methylation analysis of the *IG-DMR1* of the *DLK1-MEG3* locus in human HCC. A) Gain of bi-allelic expression is accompanied by substantial loss of methylation. B) Tumour samples displaying retention of the original imprinting pattern by strict mono-allelic expression of the same allele as in the adjacent non-cancerous liver tissue show only minor variations in DNA methylation. C) Tumour samples demonstrating allelic switching (i.e., mono-allelic expression from the normally silenced imprinted allele) show variable changes of DNA methylation patterns to an intermediate degree. The numbers in the lower left corner of each bisulfite sequencing panel are the methylation values for the region according to pyrosequencing. “G” and “A” denote the sequence at SNP Rs12437020, which allows in heterozygous samples the differentiation of the two alleles. For the calculation of the statistical significance of the changes in DNA methylation the methylation level for each clone was calculated by computing the fraction of methylated CpG sites. From these values a mean methylation level of the tumor and adjacent liver tissue sample, respectively, was calculated. These two mean values were compared using Mann-Whitney-U-test and the p-values are depicted below the schematic representation of the bisulfite sequencing results.

The two samples displaying unequivocal gain of bi-allelic expression (#20, #21) indicating loss of imprinting control show substantial loss of methylation in the IG-DMR as analysed by bisulfite sequencing. Despite the absence of informative SNPs the loss of methylation in sample #20 and #21 is evident (Fig. 7 A). For comparison the methylation patterns of two samples (#4 and #9) displaying unperturbed imprinting control with retention of mono-allelic expression in the tumour cells from the same allele as in the adjacent liver tissue were analysed (Fig. 7 B). No changes in DNA methylation are discernible. The samples displaying allelic switching (#8, #15, #16, #17, #25, #26 #30, #31) indicating unstable imprinting control show gain or loss of DNA methylation at the *IG-DMR1* to an intermediate extent (Fig. 7 C). Gain or loss of methylation at IG-DMR1 is the most recurrent alteration associated with allelic switching (see figure S9).

### Relationship of imprinting deregulation to clinical course

Despite the limited number of informative patient samples available the relationship between the above described deregulation of the *DLK1-MEG3* imprinting locus and the histopathological characteristics as well as the clinical course were analysed.

The two patient showing gain of bi-allelic expression both died within very short time after diagnosis (after 1 and 8.5 months, respectively). Global loss of DNA methylation (Figure 5) correlates significantly with tumour size (>5 cm, p = 0.029), an indicator of advanced disease. However, future large collaborative studies are necessary in order to figure out in more detail the relationship between loss of imprinting and unfavourable course of disease in human HCC.

## Discussion

Despite the fact that a relationship between imprinting defects and the development of cancer in humans is known for more than 20 years [35] this is still an underdeveloped area of research in comparison to e.g., the role of mutated oncogenes in human tumours. This is especially true for human HCC, the third leading cause of cancer mortality worldwide. Therefore, we initiated a systematic meta-analysis of the expression of all known imprinted loci in human HCC. Our study identifies loss of imprinting at the *DLK1-MEG3* locus accompanied by deregulated expression as a common epigenetic aberration in human HCC indicating widespread epigenetic instability in this human malignancy.

Allelic switching turned out to be a frequent phenomenon in human HCC affecting 8 out of 31 informative cases (26%). This loss of proper imprinting control remains undetected in most studies, because its detection requires the analysis of the corresponding normal tissue from the same patient, as performed in a pioneering study by Mai et al. [36] These authors found switched mono-allelic expression of the *p73* gene in 2 out of 12 renal cell carcinomas. However, in many studies dealing with loss of imprinting in solid tumours the imprinting status of a given gene is analysed in unaffected tissue samples from unrelated individuals and only those tumour samples which display bi-allelic expression are scored as “showing loss of imprinting”. This procedure clearly misses an unknown number of tumour samples displaying mono-allelic expression in the tumour cells but from the opposite allele compared to the adjacent normal tissue from the same patient. For myeloproliferative diseases, myelodysplastic syndrome, and overt leukaemia it is technically very difficult to obtain 100% pure cell fractions representing normal healthy haematopoiesis from the same patient. Therefore, all studies about loss of imprinting in these haematological malignancies screened by us used a series of unrelated healthy donors of bone marrow or peripheral blood for the assessment of mono-allelic expression excluding the detection of allelic switching for technical reasons. In addition, control groups with insufficient statistical power lead to incorrect conclusions about the gain or loss of imprinting [37].

For these reasons, the phenomenon of allelic switching which is an indicator of epigenetic instability is not adequately appreciated in the field and underrepresented in the literature. A study of the *p73* gene in lung cancer suggests that allelic switching is a cell type- and disease-specific phenomenon, because in contrast to the situation in renal cell carcinoma no allelic switching could be found, only gain of bi-allelic expression in 5 out of 21 cases. [38]

The absence of any aberration in DNA methylation in the *DLK1-MEG3* locus in altogether 15 benign liver tumours (figure S5) shows that in hepatocytes epigenetic instability is associated with the process of malignant transformation and not enhanced proliferation per se. In future studies the occurrence of these imprinting defects have to be analysed in dysplastic nodules of the liver. However, these lesions are quite rare, often very small, and cannot always be easily distinguished from well-differentiated HCC [39].

The data shown in Figure 2 show that not only loss of methylation but also substantial gain of methylation of DMRs can be observed in primary tumour specimens. Since this occurs in the context of global loss of methylation (see Figure 2 and 5) this is in line with previous publications showing that global loss of methylation and gain of methylation at specific sites are two independent processes in human HCC [40].

Several published studies and our own re-analysis of publicly available data sets confirmed allelic imbalances at 14q32 as a rare alteration in human HCC [41], [42]. Also all samples displaying allelic switching are heterozygous with the expected 50∶50 ratio as measured by quantitative pyrosequencing (see Figure S8). Therefore, allelic imbalance is very likely not the molecular mechanisms explaining the occurrence of allelic-switching. The most consistent feature in 7 out of 8 cases displaying allelic switching is an aberrant DNA methylation at *IG-DMR1* (loss of methylation in 4 cases, gain in 3 cases). However, since strong decrease in methylation of *IG-DMR1* is associated with gain of bi-allelic expression in case #20 and #21, additional molecular mechanisms have to be invoked.

The up-regulation of *DLK1* mRNA expression after reduction of methylation in HLE cells after *DNMT1* knock down (Fig. 4 B) seems to contradict the association between an increase in methylation of the DMRs and *DLK1* up-regulation observed in primary specimens (Fig. 3). This discrepancy is most probably due to the fact that the *DLK1* promoter is heavily methylated in HLE cells but rarely methylated in primary human HCC specimens (figure S2 F). Therefore, the activating effect of the demethylation of the *DLK1* promoter (see Figure 4 C), right panel) obviously overrides the repressing effect of demethylation of *IG-DMR1* and -*2* in HLE cells, thereby uncoupling the correlation between *IG-DMR* methylation and *DLK1* mRNA expression in these cells.

At the start of this project only a single publication could be identified dealing with *DLK1-MEG3* locus in primary human HCC samples. However, Huang et al. [15] concentrate in their study of the *DLK1-MEG3* locus in human HCC nearly exclusively on *DLK1*. While studying *DLK1* expression using RT-PCR and immunohistochemistry in two independent and fairly large cohorts (82 and 88 cases, respectively) they measured the *MEG3* RNA expression only in a small subset of samples (n = 10) and did not find any alteration. All additional experiments addressing bi-allelic expression and alterations of DNA methylation patterns deal only with *DLK1* in very small groups: They found mono-allelic *DLK1* expression in 3 informative cases, concluding that loss of imprinting does not play a role, and analysed the methylation of the *DLK1* promoter and the *IG-DMR* in 6 cases (2 showing loss of methylation, 2 showing gain of methylation).

Braconi et al. [14] addressed the involvement of altered DNA methylation patterns at the *DLK1-MEG3* locus indirectly by treating cells with the *DNMT1* inhibitor aza-cytidine (resulting in *MEG3* up-regulation in the HCC lines HepG2 and Huh-7 but not PLC/PRF-5) or knocking down DNMT1 and DNMT3b using an siRNA approach (resulting in a 1.5-fold induction of expression in HepG2 cells). In addition, they analysed the methylation of the *MEG3-DMR* in 11 HCC specimens using the MSP protocol published by Benetatos et al. [43] providing only very limited primary data for independent evaluation. Using the very same primer set published by Benetatos et al. we tried to correlate the quantitative pyrosequencing data with this MSP approach in our much larger cohort. In Figure S10 it is documented that it is not possible to detect the gains and losses of methylation identified by pyrosequencing in our study using the MSP protocol used by Benetatos et al. [43] and Braconi et al. [14].

The recently published study by Luk et al. about the *DLK1-DIO3* locus in HCC [44] concentrates nearly exclusively on the expression of microRNAs contained within this genomic region. Analyses of the DNA methylation patterns and/or the allelic-specific expression were not performed. Only the overexpression of *DLK1* mRNA in a small subset of samples is reported, without any reference to the expression of *MEG3*.

In contrast to our results shown in Figure 3 Khoury et al. could not show an inverse correlation of *MEG3* and *DLK1* expression in human acute myeloid leukaemia. [45] This shows again the cell-type specificity of imprint patterns and their deregulation in human malignancies.

In conclusion, this study demonstrates frequent epigenetic deregulation of the imprinted *DLK1*/MEG3 locus in human HCC leading to aberrant expression of *MEG3* RNA and *DLK1* mRNA. Altogether, 32% of informative samples displayed epigenetic instability rendering it one of the most frequent molecular defects in human HCC. This widespread occurrence of instability of DNA methylation patterns has to be considered for any therapeutic approach interfering with the establishment or maintenance of DNA methylation patterns (e.g. by DNMT inhibitors). On the other hand, it might serve as a useful predictive biomarker for epigenetic therapy selecting those patients most probably to respond with minimal unwanted side-effects and for monitoring response to therapy. In addition, we identified allelic switching, i.e., strict mono-allelic expression in tumour cells from the opposite allele compared to the corresponding normal tissue, as a frequent but under most circumstances overlooked indicator of epigenetic instability, which is much more prevalent than currently perceived.

## Materials and Methods

### Patient samples and cell lines

All primary patient samples were retrieved from the archive of the Institute of Pathology, Hanover Medical School (Germany) and analysed anonymously. The local Ethics committee (“Ethik-Kommission der Medizinischen Hochschule Hannover”, head: Prof. Dr. Tröger) exempted this study from review because all specimens under study were retrieved anonymously and retrospectively (left-over samples from diagnostic procedures) and waived the need for consent due to the fact the samples received were anonymous. Tumour cell content was determined to be greater than 70% using representative H&E sections.

Seven HCC cell lines (HLE, HLF, Huh7, HepG2, Hep3B, SNU182, and SNU387) and two immortalized hepatocyte lines (THLE2 and THLE3) were purchased from American Tissue Culture Collection (Rockville, MD, USA) and cultivated according to the supplier's recommendation.

### DNA and RNA isolation

DNA was isolated by digestion with proteinase K (Merck, Darmstadt, Germany) followed by phenol/chloroform extraction from a total of 40 HCC, 10 HCA, 5 FNH, and 5 healthy liver fresh-frozen specimens (Table S3). Adjacent liver tissue from the same patient was available for 34 HCC, 8 HCA, and 2 FNH, respectively.

Total RNA from fresh frozen biopsies was extracted using TRIZOL™ reagent (Invitrogen, Darmstadt, Germany) following the protocol supplied by the manufacturer.

### *In silico* screening of deregulated imprinted genes in HCC

To identify deregulation of imprinted genes in HCC, a total of 223 human imprinted genes listed at Geneimprint website (http://www.geneimprint.com/) and contained within ‘A Catalogue of Parent Origin Effects’ of the Otago University Dunedine, New Zealand (http://igc.otago.ac.nz/home.html) were screened using the microarray database Oncomine (https://www.oncomine.org/resource/login.html). Latest check of availability of all three web pages: 13^th^ September 2012. Table S1 contains all studies used for meta-analysis.

### Bisulfit conversion and methylation analysis

Bisulfit treatment of genomic DNAs was performed as described before [46]. Approximately 25 ng of converted DNA was amplified and used for pyrosequencing analysis as described [47]. All primers for PCR and pyrosequencing are available at Table S5. The methylation levels were computed by calculating the mean methylation level of all individual CpG sites covered by the corresponding pyrosequencing assay. At least two independent measurements were performed. Tumour samples were classified as “hypermethylated”, “hypomethylated”, and “normomethylated” if their methylation values were above, below, or within, respectively, the following range: mean+/−2× std of all adjacent liver tissue samples.

For allele-specific methylation analysis converted DNA was amplified and the PCR products were inserted into plasmid vector using TOPO-TA™ Cloning Kit (Invitrogen, Darmstadt, Germany). Individual clones of the PCR product were sequenced with GenomeLab™ Genetic Analysis System (Beckman Coulter, Brea, CA) using vector primers and GenomeLab™ DTCS Quick Start Kit (Beckman Coulter, Krefeld, Germany). For every sample at least 8 clones were sequenced.

### Reverse transcription and quantitative Real Time PCR (qRT-PCR)

Reverse transcription (RT) was performed in total volume of 20 µl using 1 µg purified RNA with High Capacity cDNA Reverse Transcription Kit (Invitrogen, Darmstadt, Germany) according to the manufacture's protocol. For *MEG3* and *DLK1* expression analysis qRT-PCR was performed in the ABI Prism 7500 Sequence Detection System using TaqMan Gene Expression Assays™ (Invitrogen, Darmstadt, Germany) following the manufacture's protocol and *βGUS* and *GAPDH* as reference genes.

### Allele-specific gene expression analysis

To evaluate allele-specific expression of *MEG3* and *DLK1* in primary HCC samples and HCC cell lines, polymorphisms at exonic regions showing parent-specific expression in MEG3 (Rs8013873) [31] and DLK1 (Rs1802710) [29] were analysed using pyrosequencing technology as described [48]. In heterozygous samples, further SNP analysis of the corresponding cDNA was carried out. Primers for PCR and pyrosequencing for SNP analysis are available in Table S4.

### DNMT1 knockdown

*DNMT1* knockdown in HLE cells was performed using pre-designed pools of four siRNA targeting *DNMT1* (ON-TARGET plus SMARTpool, Dharmacon/Thermo Scientific, London, UK) following the manufacturer's protocol. In brief, 2×10^4^ cells in 500 µl complete medium were seeded together with 100 µl of a previously prepared mixture containing 50 or 100 nM siRNA/well, Lipofectamine™ RNAiMAX (Invitrogen, Darmstadt, Germany), and Opti-MEM (Gibco-Invitrogen, Darmstadt, Germany) assembled sequentially as per manufacture's recommendation in individual wells of a 24-well plate. Medium containing transfection reagent was replaced with fresh medium after 24 h and repeated transfection was performed after 48 h from this point onward. After three times sequential transfections, cells were harvested for DNA, RNA, and protein extractions. Two scramble siRNAs (AllStars Negative Control siRNA, Qiagen, Hilden, Germany and Riboxx® control-N1, Riboxx, Dresden, Germany) were also included in the experiments as negative controls.

### Western Blot analysis

Following protein concentration analysis with Bradford (Pierce, Bonn, Germany), 15 µg of total proteins from cultured cell lines after siRNA treatments were subjected to electrophoresis in 10% pre-cast SDS-polyacrylamide gels (Bio-RAD, München, Germany) and then transferred onto Hybrid-P polyvinylidene difluoride (PVDF) membrane (Amersham Biosciences, Freiburg, Germany). After blocking with 5% skim milk in phosphate buffer saline-tween, the membranes were incubated with primary and followed by secondary antibodies. The signals were subsequently detected using enhanced chemilumescent (ECL) (Pierce, Bonn, Germany). Antibodies used were mouse monoclonal anti-DNMT1 antibody (IMG-261A clone 60B1220.1, Imgenex, San Diego, CA), mouse monoclonal anti-β-actin antibody (ab6276 clone AC-15, Abcam, UK), and anti-mouse secondary antibody HRP (R1253HRP, Acris, Herford, Germany).

### Statistical analysis

Statistical differences were calculated using the Mann-Whitney-U test. All calculations were performed using the software package GraphPad Prism (version 5.01 for Windows, La Jolla, CA, USA). p<0.05 were considered statistically significant.

## Supporting Information

Table S1 **List of microarray datasets retrieved from Oncomine.**
(DOC)

Table S2 **List of significantly up- or down-regulated imprinted loci in human HCC.**
(DOC)

Table S3 **Patient data.**
(DOC)

Table S4 **Results** **of quantitative SNP analyses of genomic DNA and cDNA.**
(DOC)

Table S5 **List of all primers used in this study.**
(DOC)

Figure S1 **DNA methylation levels at** ***IG-DMR*** **(1 and 2) and MEG3-DMR (1, 2 and 3) in a panel of human HCC cell lines (HLE, HLF, Huh7, HepG2, Hep3B, SNU182, SNU387) and immortalized hepatocytes (THLE2, THLE3).**
(TIF)

Figure S2 **DNA methylation of the** ***IG-DMR*** **1 and 2 and** ***MEG3-DMR*** **1, 2, and 3 as well as** ***DLK1*** **promoter in primary human HCC. Display of all individual quantitative measurements.**
(TIF)

Figure S3 **Reduction of DNMT1 protein (A) and mRNA (B) after siRNA-mediated knock down.**
(TIF)

Figure S4 **Correlation between** ***DLK1/MEG3*** **DMRs and global methylation (measured as** ***LINE-1*** **methylation values).**
(TIF)

Figure S5 **DNA methylation analysis of** ***DLK1/MEG3*** **imprinting locus in benign liver tumours.** *IG-DMR* 1 (A), *IG-DMR* 2 (B), *MEG3-DMR* 1(C), *MEG3-DMR* 2 (D), *MEG3-DMR* 3 (E), and *DLK1* promoter (F).
(TIF)

Figure S6 **LINE-1 methylation in HCA (n = 10) and FNH (n = 5) and in corresponding adjacent liver tissue as well as in unrelated healthy liver tissues (n = 5).**
(TIF)

Figure S7 **Sanger sequencing of cDNA confirming gain of bi-allelic expression (A) and allelic switching (B) in tumour samples.**
(TIF)

Figure S8 **Quantitative SNP analysis of the genomic and cDNAs from the tumour specimens displaying allelic switching for** ***MEG3*** **(A) and** ***DLK1*** **(B–F).**
(TIF)

Figure S9 **Bisulfite sequencing of** ***MEG3*** **DMR1 – 3 for the 8 tumours displaying allelic switching.**
(TIF)

Figure S10 **Methylation analysis using MSP with primers described by Benetatos et al.** [**43**] **, and also used by Braconi et al.** [**14**] **.**
(DOC)
