# Supplementary material for: Loss of Imprinting and Allelic Switching at the DLK1-MEG3 Locus in Human Hepatocellular Carcinoma
Source: PLoS One. 2012 Nov 8;7(11):e49462. doi: 10.1371/journal.pone.0049462 (PMC3493531; doi:10.1371/journal.pone.0049462)
Supplement: Table S2 — List of significantly up- or down-regulated imprinted loci in human HCC. (DOC) [file pone.0049462.s002.doc]

**Supplementary Table S2**

List of imprinted genes that are differentially expressed in Oncomine datasets

| **Nr** | **Imprinted genes** | **Downregulated** | **p value** | **Fold change** | **Upregulated** | **p value** | **Fold change** |
| --- | --- | --- | --- | --- | --- | --- | --- |
| **1** | DIRAS3/ARHI | Ref. 3,9,10,14 | 0.012-<0.0001 | 1.2 -7.8 |  |  |  |
| **2** | PTPN14 |  |  |  | Ref. 3, 14 | 0.012-0.034 | 1.13-1.73 |
| **3** | OBSCN |  |  |  | Ref. 14 | 0.045 | 1.1 |
| **4** | ZIC1 | Ref.9 | <0.0001 | 1.7 |  |  |  |
| **5** | FAM50B | Ref. 9, 14 | 0.02-<0.0001 | 1.35-1.5 |  |  |  |
| **6** | IGF2R | Ref.9 | <0.0001 | 1.4 | Ref. 3, 10 | <0.0001 | 1.17-1.3 |
| **7** | SLC22A2 | Ref.10 | 0.006 | 1.17 |  |  |  |
| **8** | HOXA3 |  |  |  | Ref 9, 14 | 0.028-<0.0001 | 1.085-3.7 |
| **9** | HOXA5 |  |  |  | Ref 3, 10, 14 | 0.04-<0.0001 | 1.15-1.56 |
| **10** | GLI3 | Ref. 9, 10,11 | 0.04-<0.0001 | 1.1-1.4 |  |  |  |
| **11** | DDC | Ref.14 | <0.0001 | 1.9 | Ref.9 | <0.0001 | 1.4 |
| **12** | TFPI2 | Ref.3, 10, 14 | <0.0001 | 1.4-9,6 |  |  |  |
| **13** | PEG10 |  |  |  | Ref. 3, 9, 10, 14 | 0.03-<0.0001 | 2.1-15.5 |
| **14** | PON2 |  |  |  | Ref. 3, 9, 10 | 0.008-<0.0001 1.24-1.57 | |
| **15** | DLGAP2 | Ref. 9, 10, 14 | <0.007 | 1.03-1.2 |  |  |  |
| **16** | PURG | Ref.10, 14 | 0.002-0.0015 | 1.05-1.048 | |  |  |
| **17** | NKAIN3 | Ref.14 | 0.011 | 1.2 |  |  |  |
| **18** | KCNK9 |  |  |  | Ref.14 | 0.06 | 1.1 |
| **19** | ABCA1 | Ref. 9, 10, 14 | 0.004-<0.0001 | 1.1-1.9 |  |  |  |
| **20** | GATA3 | Ref 3, 10 | 0.032-0.0001 | 1.3-1.7 | Ref 9, 14 | <0.0001 | 1.2-1.48 |
| **21** | PKP3 | Ref 9, 10, 14 | 0.003-<0.0001 | 1.05-1.15 |  |  |  |
| **22** | H19 | Ref.3, 14 | 0.006-<0.0001 | 2.8-13 |  |  |  |
| **23** | SDHD | Ref. 3, 10, 14 | 0.02-<0.0001 | 1.2-1.8 |  |  |  |
| **24** | ABCC9 | Ref. 9, 10, 14 | <0.0001 | 1.4-5.8 |  |  |  |
| **25** | SLC38A4 | Ref 3, 9, 10, 14 | 0.014-<0.0001 | 1.36-5.8 |  |  |  |
| **26** | SLC26A10 | Ref. 10 | <0.0001 | 1.12 |  |  |  |
| **27** | FERMT2 | Ref. 9, 10, 14 | <0.0001 | 1.61-2.6 | Ref. 3 | 0.004 | 1.14 |
| **28** | MEG3 | Ref. 1, 2, 3, 10, 12, 14 | 0.020-<0.0001 | 1.06-1.38 | Ref 9, 16 | 0.013-<0.0001 | 1.4-1.5 |
| **29** | NDN | Ref. 2 | <0.0001 | 2.2 |  |  |  |
| **30** | GABRB3 | Ref. 3, 9 | <0.0001 | 1.08-2.8 | Ref. 9 | <0.0001 | 1.33 |
| **31** | UBE3A | Ref. 3, 9, 10, 14 | 0.022-<0.0001 | 1.038-1.245 | Ref. 10 |  |  |
| **32** | FOXF1 | Ref. 9 | <0.0001 | 1.43 |  |  |  |
| **33** | PPAP2C | Ref. 10 | <0.0001 | 1.4 | Ref. 9, 14 | <0.0001 | 1.2 |
| **34** | PEG3 | Ref. 9, 14 | <0.0001 | 2.6-3.3 | Ref.10 | <0.0001 | 1.67 |
| **35** | C20orf82 | Ref. 14 | <0.0001 | 3.5 |  |  |  |
| **36** | ISM1 | Ref. 14 | <0.0001 | 3.5 |  |  |  |
| **37** | NNAT | Ref. 10, 14 | <0.0001 | 1.12-1.15 |  |  |  |
| **38** | XIST |  |  |  | Ref. 3, 14 | 0.02-<0.0001 | 2.78-13 |

References:

1. Archer KJ, Mas VR, David K, Maluf DG, Bornstein K, Fisher RA. Identifying genes for establishing a multigenic test for hepatocellular carcinoma surveillance in hepatitis C virus-positive cirrhotic patients. Cancer Epidemiol Biomarkers Prev. 2009;18(11):2929-32.
2. Bittner M. Not published. International Genomics Consortium Expression Project for Oncology.
3. Chen X, Cheung ST, So S, Fan ST, Barry C, Higgins J, Lai KM, Ji J, Dudoit S, Ng IO, Van De Rijn M, Botstein D, Brown PO. Gene expression patterns in human liver cancers. Mol Biol Cell 2002;13:1929-39
4. Chiang DY, Villanueva A, Hoshida Y., et al. Focal Gains of VEGFA and Molecular Classification of Hepatocellular Carcinoma. Cancer Res 2008;68(16):6779-87.
5. Gyorffy B, Surowiak P, Kiesslich O, Denkert C, Schafer R, Dietel M, Lage H . Gene expression profiling of 30 cancer cell lines predicts resistance towards 11 anticancer drugs at clinically achieved concentrations. Int J Cancer. 2006;118(7):1699-712.
6. Iizuka N, Oka M, Yamada-Okabe H, Nishida M, Maeda Y, Mori N, Takao T, Tamesa T, Tangoku A, Tabuchi H, Hamada K, Nakayama H, Ishitsuka H, Miyamoto T, Hirabayashi A, Uchimura S, Hamamoto Y. Oligonucleotide microarray for prediction of early intrahepatic recurrence of hepatocellular carcinoma after curative resection. Lancet. 2003;361(9361):923-9.
7. Jia HL, Ye QH, Qin LX, Budhu A, Forgues M, Chen Y, Liu YK, Sun HC, Wang L, Lu HZ, Shen F, Tang ZY, Wang XW. Gene expression profiling reveals potential biomarkers of human hepatocellular carcinoma. Clin Cancer Res. 2007. 15;13(4):1133-9.
8. Liao YL, Sun YM, Chau GY, Chau YP, Lai TC, Wang JL, Horng JT, Hsiao M, Tsou AP. Identification of SOX4 target genes using phylogenetic footprinting-based prediction from expression microarrays suggests that overexpression of SOX4 potentiates metastasis in hepatocellular carcinoma. Oncogene. 2008 18;27(42):5578-89.
9. Mas VR, Maluf DG, Archer KJ, Yanek K, Kong X, Kulik L, Freise CE, Olthoff KM, Ghobrial RM, McIver P, Fisher R. Genes involved in viral carcinogenesis and tumor initiation in Hepatitis C Virus-induced Hepatocellular Carcinoma. Mol Med. 2009;15(3-4):85-94.
10. Roessler S, Jia HL, Budhu A, Forgues M, Ye QH, Lee JS, Thorgeirsson SS, Sun Z, Tang ZY, Qin LX, Wang XW. A unique metastasis gene signature enables prediction of tumor relapse in early-stage hepatocellular carcinoma patients. Cancer Res. 2010;70(24):10202-12.
11. Su AI, Welsh JB, Sapinoso LM, Kern SG, Dimitrov P, Lapp H, Schultz PG, Powell SM, Moskaluk CA, Frierson HF Jr, Hampton GM. Molecular classification of human carcinomas by use of gene expression signatures. Cancer Res 2001.
12. Woo HG, Lee JH, Yoon JH, Kim CY, Lee HS, Jang JJ, Yi NJ, Suh KS, Lee KU, Park ES, Thorgeirsson SS, Kim YJ. Identification of a cholangiocarcinoma-like gene expression trait in hepatocellular carcinoma. Cancer Res. 2010.15;70(8):3034-41
13. Wooster R. Unpublished data. Transcript profiling of cancer cell line panel. http://www.ncbi.nlm.nih.gov/geo/query/acc.cgi?acc=GSE15765
14. Wurmbach E, Chen YB, Khitrov G, Zhang W, Roayaie S, Schwartz M, Fiel I, Thung S, Mazzaferro V, Bruix J, Bottinger E, Friedman S, Waxman S, Llovet JM. Genome-wide molecular profiles of HCV-induced dysplasia and hepatocellular carcinoma. Hepatology 2007;45(4):938-47.
15. Ye QH, Qin LX, Forgues M, He P, Kim JW, Peng AC, Simon R, Li Y, Robles AI, Chen Y, Ma ZC, Wu ZQ, Ye SL, Liu YK, Tang ZY, Wang XW. Predicting hepatitis B virus- positive metastatic hepatocellular carcinomas using gene expression profiling and supervised machine learning. Nat Med. 2003;9(4):416-23.
16. Yu K, Ganesan K, Tan LK, Laban M, Wu J, Zhao XD, Li H, Leung CH, Zhu Y, Wei CL, Hooi SC, Miller L, Tan P. A precisely regulated gene expression cassette potently modulates metastasis and survival in multiple solid cancers. PLoS Genet 2008.
