# Supplementary material for: Loss of Imprinting and Allelic Switching at the DLK1-MEG3 Locus in Human Hepatocellular Carcinoma
Source: PLoS One. 2012 Nov 8;7(11):e49462. doi: 10.1371/journal.pone.0049462 (PMC3493531; doi:10.1371/journal.pone.0049462)
Supplement: Table S3 — Patient data. (DOC) [file pone.0049462.s003.doc]

**Supplementary Table S3**

All primary specimens used in this study

HCC

| **HCC n=40** |  | **n** |
| --- | --- | --- |
| **Age** |  |  |
|  | <50y | 12 |
|  | >50y | 28 |
| **Sex** |  |  |
|  | Male | 33 |
|  | Female | 7 |
| **Etiology** |  |  |
|  | HBV | 8 |
|  | HCV | 4 |
|  | No infection | 28 |
| **Tumor differentiation** | |  |
|  | Good | 15 |
|  | Moderate | 17 |
|  | Poor | 8 |
| **Tumor size** | |  |
|  | <5cm | 20 |
|  | >5cm | 20 |
| **Stage** |  |  |
|  | I | 5 |
|  | II | 11 |
|  | III | 16 |
|  | IV | 8 |
| **Number of nodules** | |  |
|  | Unilocular | 14 |
|  | Multilocular | 26 |
| **Cirrhosis** |  |  |
|  | With Cirrhosis | 32 |
|  | Without Cirrhosis | 8 |
| **Survival** |  |  |
|  | <3 years | 18 |
|  | > 3years | 17 |
|  | No information | 2 |
|  | diagnosed <2y ago | 3 |

HCA and FNH

|  |  | **HCA (n=10)** | **FNH n=5** |
| --- | --- | --- | --- |
| **Age** |  |  |  |
|  | <50y | 10 | 4 |
|  | >50y | 0 | 1 |
| **Sex** |  |  |  |
|  | Male | 1 | 2 |
|  | Female | 9 | 3 |
| **Tumor size** | |  |  |
|  | <5cm | 4 | 4 |
|  | >5cm | 6 | 1 |
| **Number of nodules** | |  |  |
|  | Unilocular | 9 | 5 |
|  | Multilocular | 1 | 1 |
