# Supplementary material for: Loss of Imprinting and Allelic Switching at the DLK1-MEG3 Locus in Human Hepatocellular Carcinoma
Source: PLoS One. 2012 Nov 8;7(11):e49462. doi: 10.1371/journal.pone.0049462 (PMC3493531; doi:10.1371/journal.pone.0049462)
Supplement: Table S4 — Results of quantitative SNP analyses of genomic DNA and cDNA. (DOC) [file pone.0049462.s004.doc]

**Supplementary Table S4**

|  | **MEG3 SNP PMID 10759892** | | | | **MEG3 SNP Rs8013873** | | | | **DLK1 SNP Rs1802710** | | | |
| --- | --- | --- | --- | --- | --- | --- | --- | --- | --- | --- | --- | --- |
| **Nu** | gDNA Tu | cDNA Tu | gDNA Adj | cDNA Adj | gDNA Tu | cDNA Tu | gDNA Adj | cDNA Adj | gDNA Tu | cDNA Tu | gDNA Adj | cDNA Adj |
| **1** | CC |  |  |  | TT | TT | TT | TT | TT | TT | TT | TT |
| **2** | CG | CC | CG | CC | CC |  |  |  | TT |  |  |  |
| **3** | CG | CC | CG | CC | CC |  |  |  | TC | CC | TC | CC |
| **4** | CC |  |  |  | CC |  |  |  | TT |  |  |  |
| **5** | CC |  |  |  | CC |  |  |  | TC | CC | TC | CC |
| **6** | CC |  |  |  | CC |  |  |  | TC | CC | TC | CC |
| **7** | CG | CC | CG | CC | CC |  |  |  | TT |  |  |  |
| **8** | CC |  |  |  | TC | CC | TC | CC | **TC** | **CC** | **TC** | **TT** |
| **9** | CC |  |  |  | TT | TT | TT | TT | CC |  |  |  |
| **10** | CC |  |  |  | CC |  |  |  | CC |  |  |  |
| **11** | CG | CC | CG | CC | CC |  |  |  | CC |  |  |  |
| **12** | CC |  |  |  | CC |  |  |  | CC |  |  |  |
| **13** | CC |  |  |  | TC | TT | TC | TT | CC |  |  |  |
| **14** | CC |  |  |  | CC |  |  |  | TT |  |  |  |
| **15** | CC |  |  |  | CC |  |  |  | TC | CC | TC | TT |
| **16** | CC |  |  |  | CC |  |  |  | TC | CC | TC | TT |
| **17** | CC |  |  |  | CC |  |  |  | TC | CC | TC | TT |
| **18** | CC |  |  |  | CC |  |  |  | TC | TT | TC | TT |
| **19** | CC |  |  |  | CC |  |  |  | TT |  |  |  |
| **20** | CC |  |  |  | CC |  |  |  | TC | TC | TC | CC |
| **21** | CC |  |  |  | TC | TC | TC | CC | TC | TT | TC | CC |
| **22** | CC |  |  |  | TC | CC | TC | CC | CC |  |  |  |
| **23** | CC |  |  |  | TC | CC | TC | CC | TC | TT | TC | TT |
| **24** | CC |  |  |  | CC |  |  |  | TC | TT | TC | TT |
| **25** | CC |  |  |  | TC | TT | TC | CC | CC |  |  |  |
| **26** | CC |  |  |  | TC | CC | TC | TT | CC | CC |  |  |
| **27** | CC |  |  |  | CC |  |  |  | TT |  |  |  |
| **28** | CC |  |  |  | CC |  |  |  | TT |  |  |  |
| **29** | CC |  |  |  | CC |  |  |  | TC | TT | TC | TT |
| **30** | CC |  |  |  | CC | CC |  |  | TC | CC | TC | TT |
| **31** | CC |  |  |  | CC |  |  |  | TC | CC | TC | TT |
| **32** | CG | CC | CG | CC | TC | TT | TC | TT | TC | CC | TC | CC |
| **33** | CC |  |  |  | CC |  |  |  | TC | CC | TC | CC |
| **34** | CC |  |  |  | TC | TT | TC | TT | TC | TT | TC | TT |
| **35** | CC | NS | NS | NS | TC | NS |  |  | TC | NS | NS | NS |
| **36** | CC |  | NS | NS | CC |  |  |  | TC | CC | NS | NS |
| **37** | CC |  | NS | NS | TT |  |  |  | TT |  | NS | NS |
| **38** | CC |  | NS | NS | CC |  |  |  | TC | CC | NS | NS |
| **39** | CC |  | NS | NS | CC |  |  |  | TC | CC | NS | NS |
| **40** | CC |  | NS | NS | TC | CC | TC |  | TC | TT | NS | NS |

Results of the SNP analysis for the identification of informative cases for the allele-specific expression

gDNA = genomic DNA

Tu = Tumor

Adj = adjacent liver tissue

NS = no sample available

= allele switching

= biallelic expression
