# Supplementary material for: Loss of Imprinting and Allelic Switching at the DLK1-MEG3 Locus in Human Hepatocellular Carcinoma
Source: PLoS One. 2012 Nov 8;7(11):e49462. doi: 10.1371/journal.pone.0049462 (PMC3493531; doi:10.1371/journal.pone.0049462)
Supplement: Table S5 — List of all primers used in this study. (DOC) [file pone.0049462.s005.doc]

**Supplementary Table S5**

**Primers used in this study**

| **Name** | **Forward** | **Reverse** | **Sequencing** | **[MgCl2] mM** | **T-ann [C]** |
| --- | --- | --- | --- | --- | --- |
| **Pyrosequencing** | | | | | |
| **IG-DMR (1)** | ATTATTGAATTGGGTTTGTTAGTAG | CAAAACAACTCAAATCCTTTATAAC | TTAAAATATATCAAAAAACC | 2.5 | 60 |
| **IG-DMR (2)** | TAGYGATTTGTTAATTGYGAGTG | CRAATCCATTATAACCAATTACAATACCAC | CAATTACAATACCACAAAAT | 1.5 | 60 |
| **MEG3-DMR(1)** | TTGGTTATYGGTYGTTTGAGG | AAAAAAATTCTACCRCAATACCCC | CAATACCCCTAACCRCCATAAC | 2.5 | 60 |
| **MEG3-DMR(2)** | GTATTTTGATTTTTGYGAGAGGAT | ATCCCCACACACATACCCTTT | CTTTAAAAAAACCCCAA | 2.5 | 60 |
| **MEG3-DMR(3)** | GTTYGTATTTTTYGATGGATGTT | CACCCTATAATCRCRAATACTTTT | ATAACTAACCRTCCTCAAAC | 2.5 | 60 |
| **DLK1 promoter** | TTYGGGATTTTAGYGATAAGTGTT | CRTTTAATACACRTTCCCTCACAC | GTTCCCTCACACTATACAAC | 1.5 | 60 |
| **LINE1** | TTTTATTAGGGAGTGTTAGATA | AAAAAAAAACTCCCTAACCCC | CCAAATAAAACAATACCTC | 1.5 | 60 |
| **MEG3 SNP ASE** | CATCCTGCTGGCAACTCC | TTCCCCCAGAAAAGGATAGG | ACTGAATCACCAAAGGCA | 2.5 | 65 |
| **Rs8013873** | TGTTTCCAGCAGGTTCCCAGTGC | GAGGATGCTTGGCAGGAGATGGA | AGGTTCCCAGTGCCC | 2.5 | 65 |
| **Rs1802710** | ACCCATGCGAGAACGACGGC | GATGAAGCCGGCTGGGCACC | CGACGGCGTCTGCACTGA | 2.5 | 65 |
|  |  |  |  |  |  |
| **Bisulfit sequencing** | | | | | |
| **IG-DMR (1)** | TTTTATTATTGAATTGGGTTTGTTAGT | ACAATTCCTACTACAAAATTTCAACA |  | 1.5 | 57 |
| **MEG3DMR1-3** | GTTTATATTTGGGAATTAGTTATGT | AAAAAAATTCTACCRCAATACCCC |  | 2.5 | 60 |
| **MEG3DMR2** | GTATTTTGATTTTTGYGAGAGGAT | ATCCCCACACACATACCCTTT |  | 2.5 | 60 |
|  |  |  |  |  |  |
| **MS-PCR** |  |  |  |  |  |
| **MEG3p M** | GTTAGTAATCGGGTTTGTCGGC | AATCATAACTCCGAACACCCGCG |  | 1.5 | 70/65/60 |
| **MEG3p U** | GAGGATGGTTAGTTATTGGGGT | CCACCATAACCAACACCCTATAATCACA |  | 1.5 | 70/65/60 |

Y=C/T

R=T/A
