# Supplementary figures and images for: Loss of Imprinting and Allelic Switching at the DLK1-MEG3 Locus in Human Hepatocellular Carcinoma
Source: PLoS One. 2012 Nov 8;7(11):e49462. doi: 10.1371/journal.pone.0049462 (PMC3493531; doi:10.1371/journal.pone.0049462)

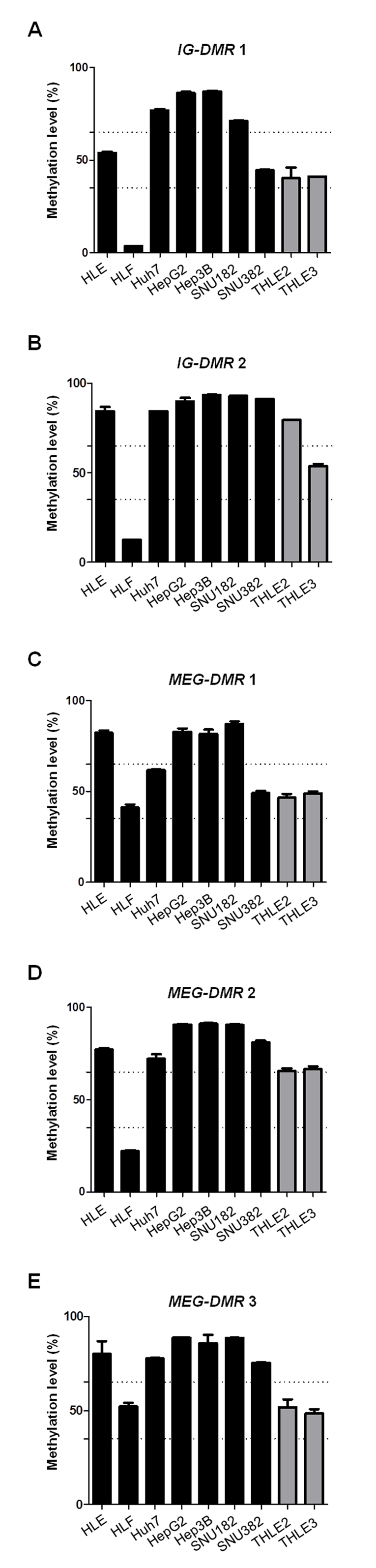

Supplement: Figure S1 — DNA methylation levels at IG-DMR (1 and 2) and MEG3-DMR (1, 2 and 3) in a panel of human HCC cell lines (HLE, HLF, Huh7, HepG2, Hep3B, SNU182, SNU387) and immortalized hepatocytes (THLE2, THLE3). (TIF) [file pone.0049462.s006.tif]

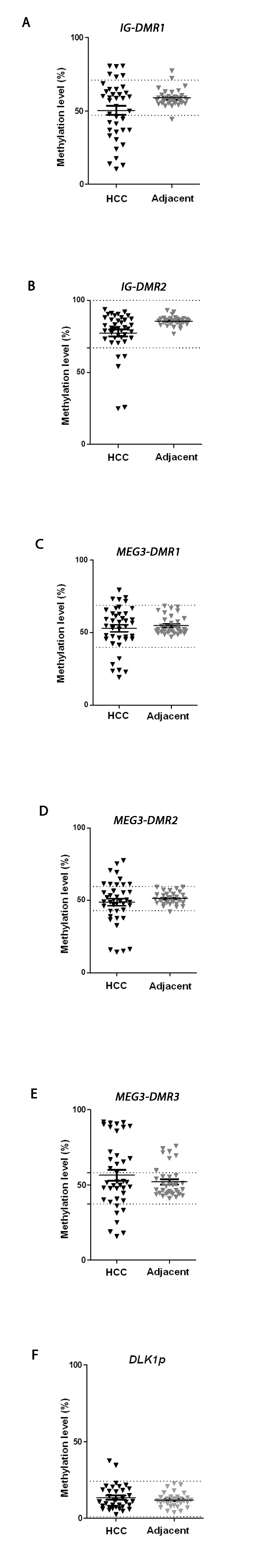

Supplement: Figure S2 — DNA methylation of the IG-DMR 1 and 2 and MEG3-DMR 1, 2, and 3 as well as DLK1 promoter in primary human HCC. Display of all individual quantitative measurements. (TIF) [file pone.0049462.s007.tif]

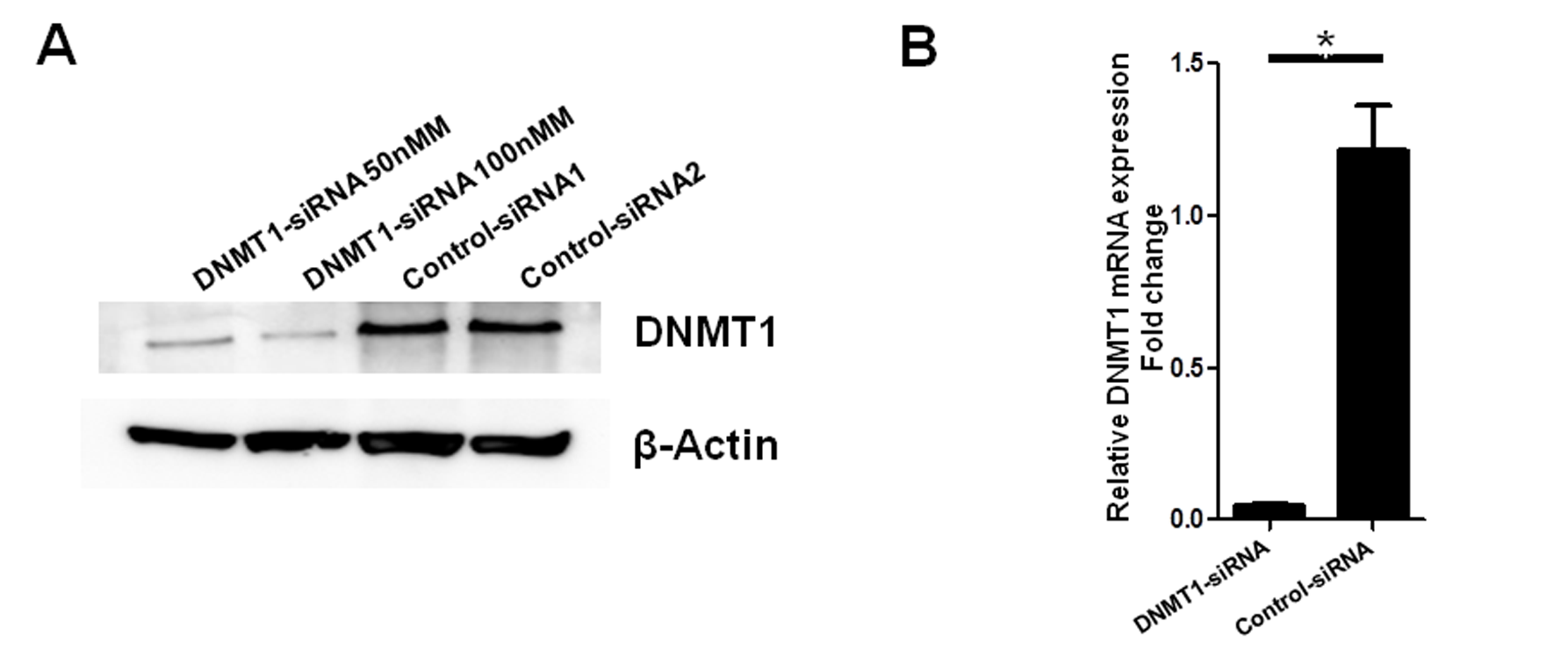

Supplement: Figure S3 — Reduction of DNMT1 protein (A) and mRNA (B) after siRNA-mediated knock down. (TIF) [file pone.0049462.s008.tif]

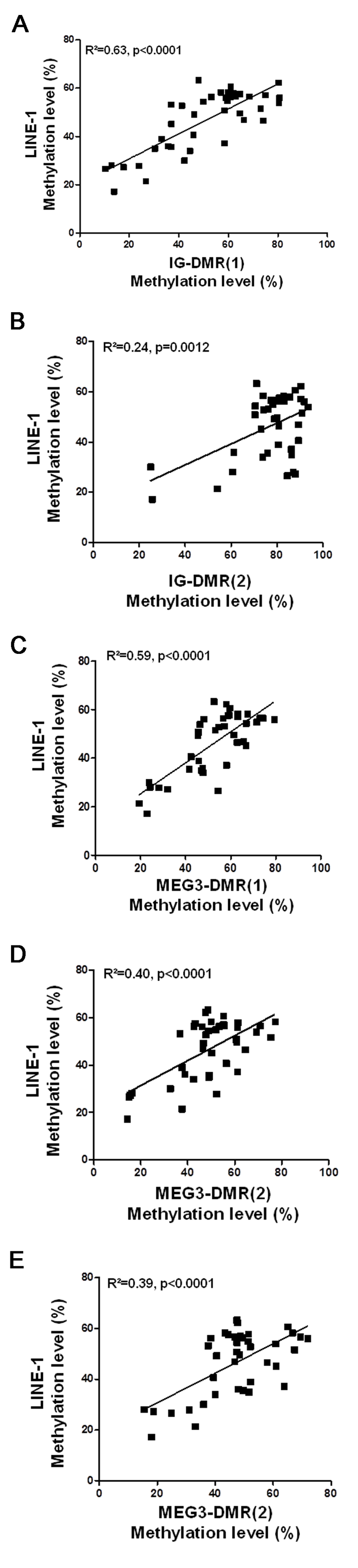

Supplement: Figure S4 — Correlation between DLK1/MEG3 DMRs and global methylation (measured as LINE-1 methylation values). (TIF) [file pone.0049462.s009.tif]

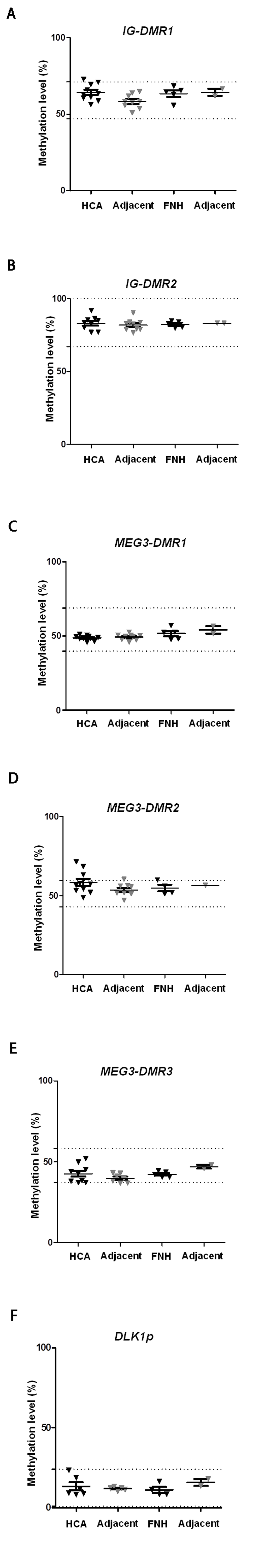

Supplement: Figure S5 — DNA methylation analysis of DLK1/MEG3 imprinting locus in benign liver tumours. IG-DMR 1 (A), IG-DMR 2 (B), MEG3-DMR 1(C), MEG3-DMR 2 (D), MEG3-DMR 3 (E), and DLK1 promoter (F). (TIF) [file pone.0049462.s010.tif]

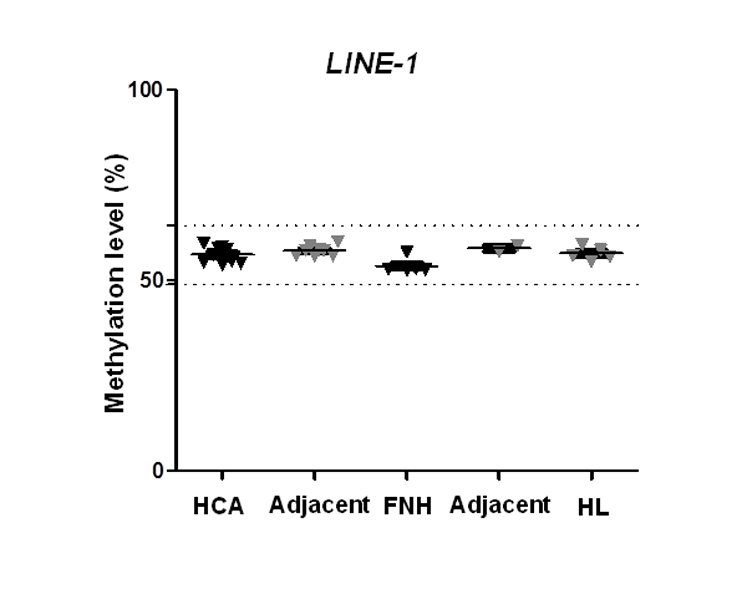

Supplement: Figure S6 — LINE-1 methylation in HCA (n = 10) and FNH (n = 5) and in corresponding adjacent liver tissue as well as in unrelated healthy liver tissues (n = 5). (TIF) [file pone.0049462.s011.tif]

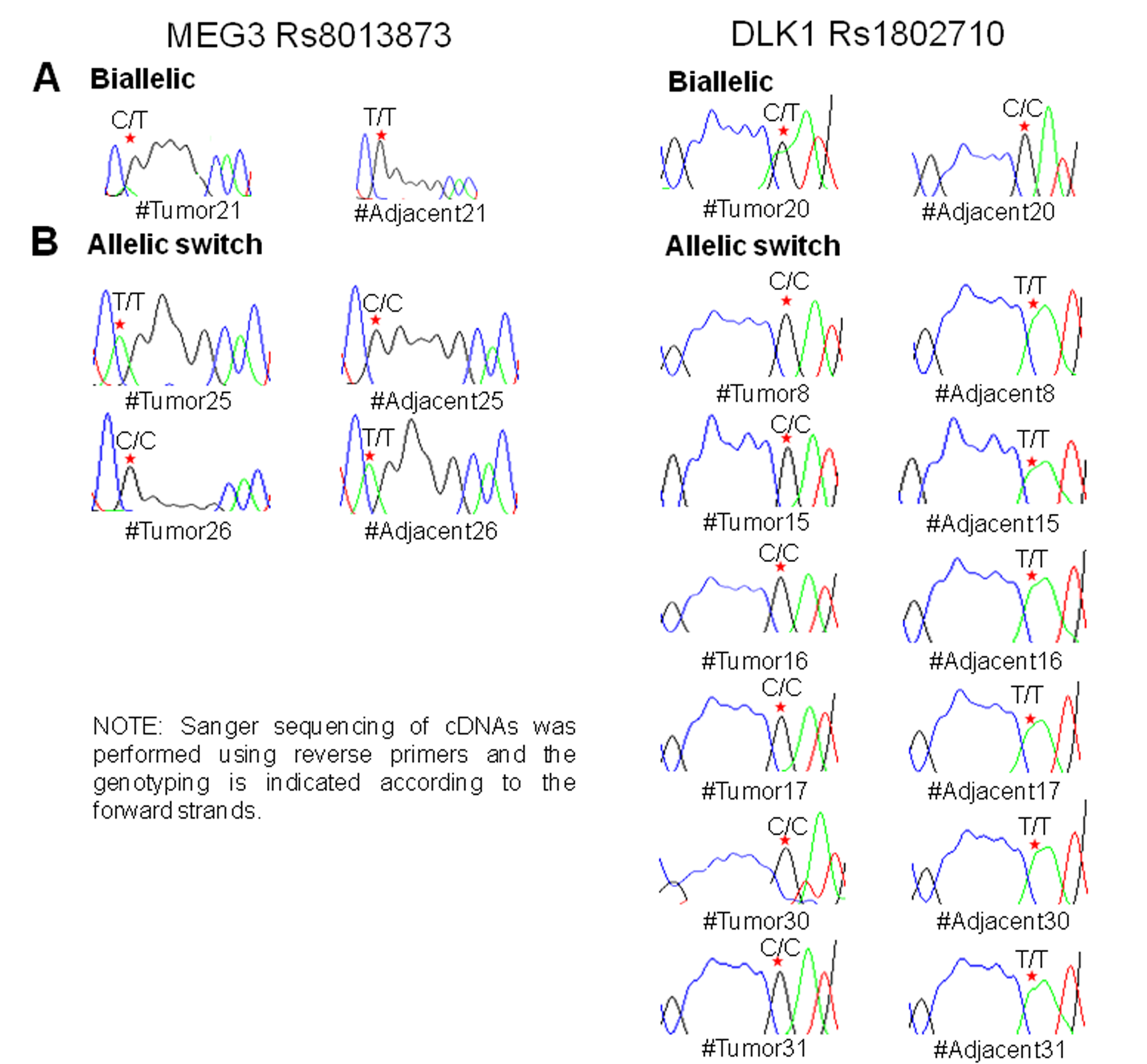

Supplement: Figure S7 — Sanger sequencing of cDNA confirming gain of bi-allelic expression (A) and allelic switching (B) in tumour samples. (TIF) [file pone.0049462.s012.tif]

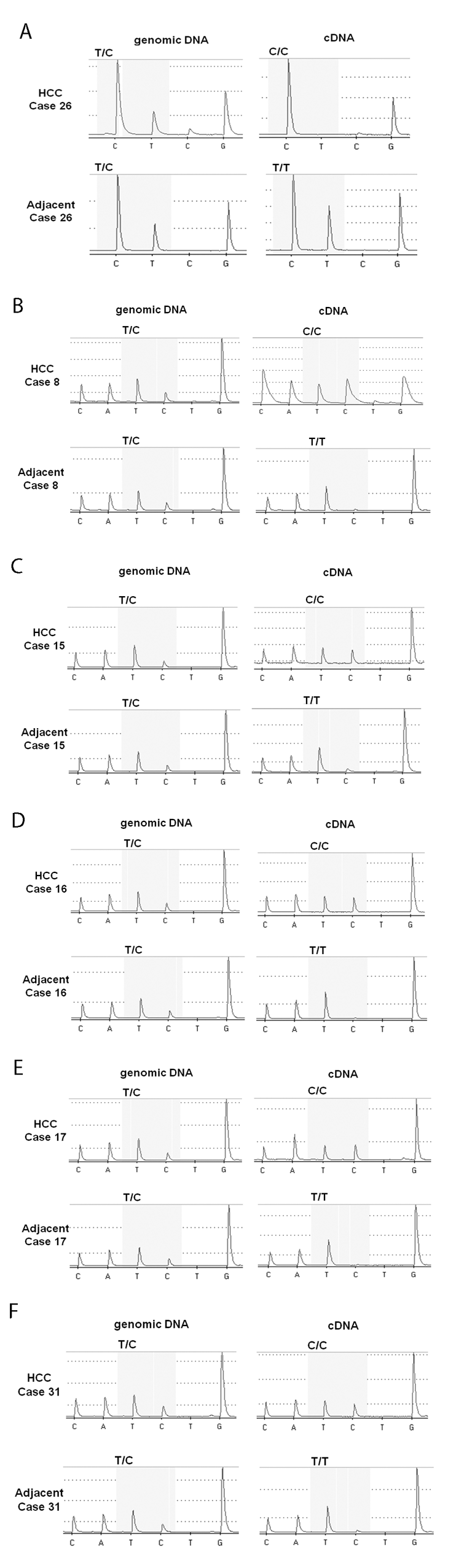

Supplement: Figure S8 — Quantitative SNP analysis of the genomic and cDNAs from the tumour specimens displaying allelic switching for MEG3 (A) and DLK1 (B–F). (TIF) [file pone.0049462.s013.tif]

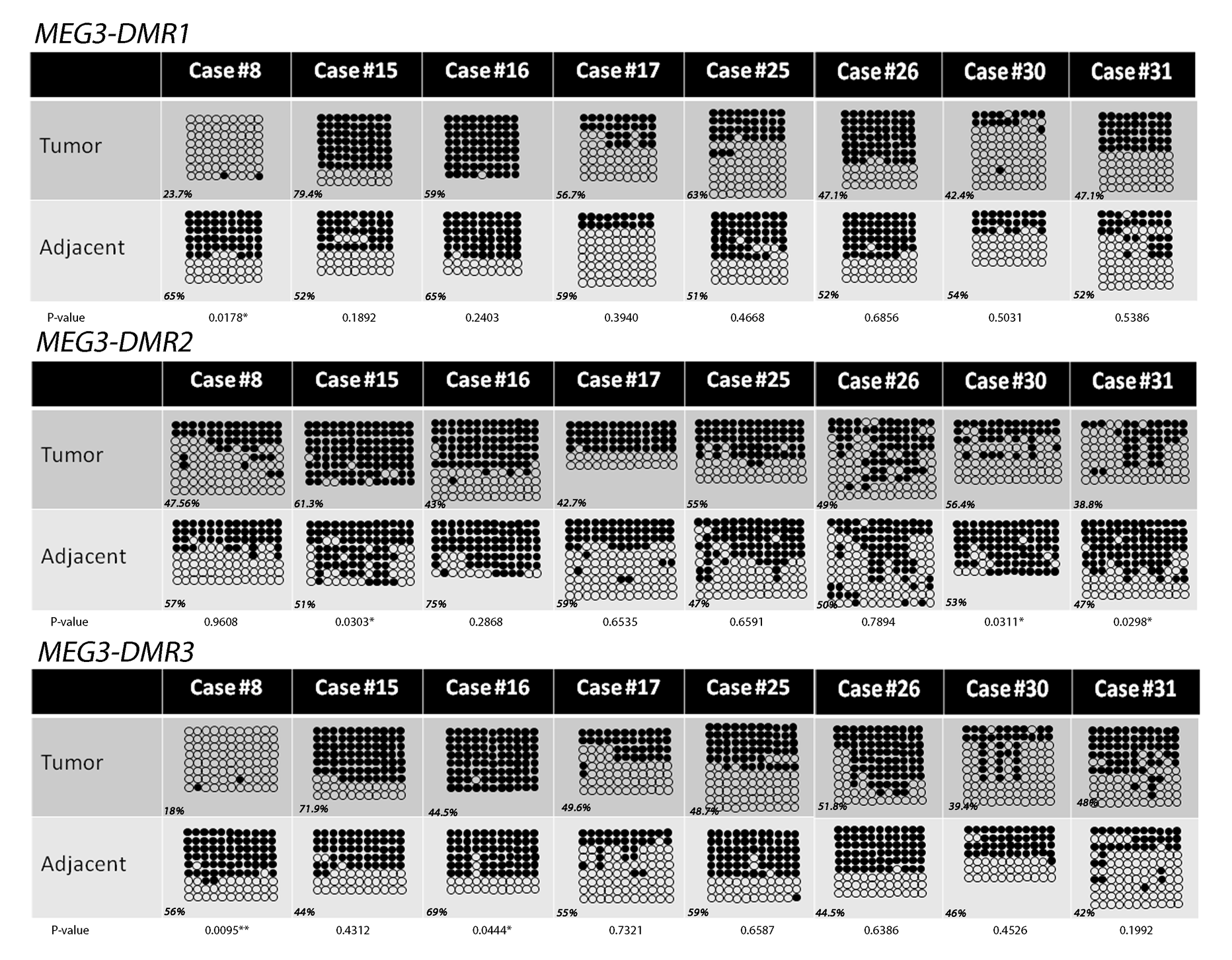

Supplement: Figure S9 — Bisulfite sequencing of MEG3 DMR1 – 3 for the 8 tumours displaying allelic switching. (TIF) [file pone.0049462.s014.tif]

**Supplement Figure 10**

HCC cell lines


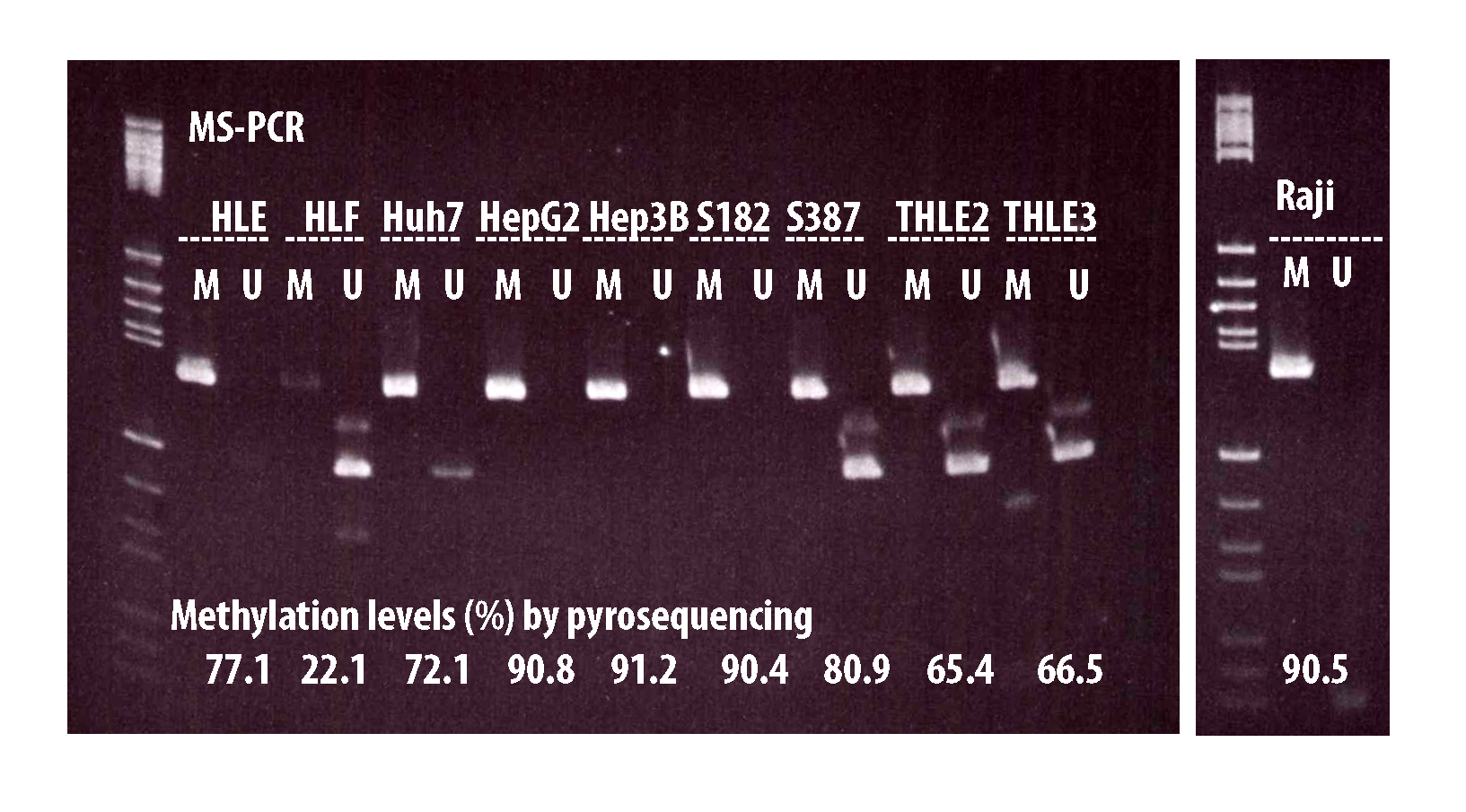


Primary HCC tumor and adjacent liver tissues


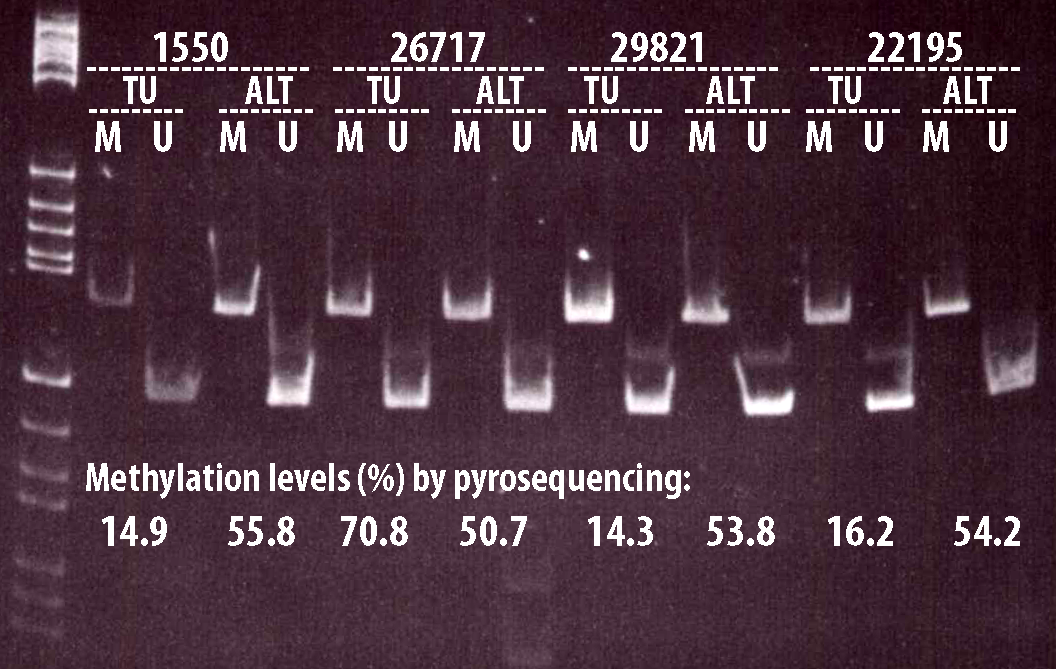


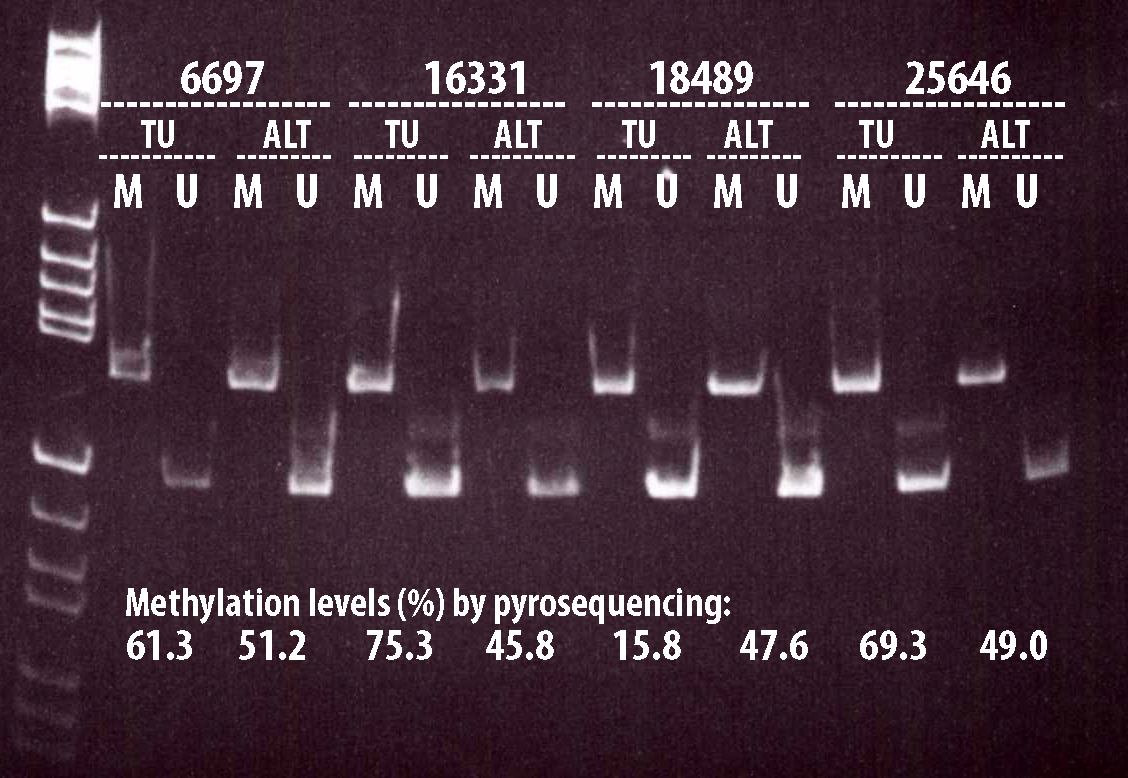

Supplement: Figure S10 — Methylation analysis using MSP with primers described by Benetatos et al. [43] , and also used by Braconi et al. [14] . (DOC) [file pone.0049462.s015.doc]
